# The *Drosophila* CLAMP protein associates with diverse proteins on chromatin

**DOI:** 10.1371/journal.pone.0189772

**Published:** 2017-12-27

**Authors:** Jennifer A. Urban, John M. Urban, Guray Kuzu, Erica N. Larschan

**Affiliations:** Department of Molecular Biology, Cellular Biology and Biochemistry, Brown University, Providence, RI, United States of America; Hirosaki University Graduate School of Medicine, JAPAN

## Abstract

Gaining new insights into gene regulation involves an in-depth understanding of protein-protein interactions on chromatin. A powerful model for studying mechanisms of gene regulation is dosage compensation, a process that targets the X-chromosome to equalize gene expression between XY males and XX females. We previously identified a zinc finger protein in *Drosophila melanogaster* that plays a sex-specific role in targeting the Male-specific lethal (MSL) dosage compensation complex to the male X-chromosome, called the Chromatin-Linked Adapter for MSL Proteins (CLAMP). More recently, we established that CLAMP has non-sex-specific roles as an essential protein that regulates chromatin accessibility at promoters genome-wide. To identify associations between CLAMP and other factors in both male and female cells, we used two complementary mass spectrometry approaches. We demonstrate that CLAMP associates with the transcriptional regulator complex Negative Elongation Factor (NELF) in both sexes and determine that CLAMP reduces NELF recruitment to several target genes. In sum, we have identified many new CLAMP-associated factors and provide a resource for further study of this little understood essential protein.

## Introduction

Identification of functional chromatin-associated protein-protein interactions has been important in understanding the establishment of dosage compensation in *Drosophila melanogaster* [1]. Dosage compensation is a conserved process, which in *D*. *melanogaster* occurs by increasing transcript levels expressed from the single male X-chromosome to equal those expressed from the two female X-chromosomes [2]. While it is known that the Male-specific lethal (MSL) complex facilitates the process of *D*. *melanogaster* dosage compensation [3], the MSL complex does not include any sequence-specific DNA binding proteins with high affinity for the GA-rich *cis*-elements that target it to the male X-chromosome [4]. Therefore, the mechanism by which the MSL complex recognizes the X-chromosome remained poorly understood. Using a cell-based RNA interference approach, we recently demonstrated that a zinc finger protein is a key regulator of MSL complex recruitment to the X-chromosome [5,6], which we named Chromatin-linked adapter for MSL Proteins (CLAMP). Subsequently, we determined that CLAMP is an essential protein that binds to thousands of GA-rich sequences throughout the genome in both males and females, explaining why it was not originally identified in male-specific lethal screens [6–8].

It was previously suggested that weakly associated factors essential for MSL complex function might not be tightly associated with the core MSL complex components: MSL1, MSL2, MSL3, and MOF [1]. Two of these factors are Maleless (MLE) and the Serine/Threonine kinase JIL-1, which are associated with the MSL complex but do not co-purify with core complex components [9]. These interactions were hypothesized to be unstable when disassociated from chromatin, making them difficult to purify using traditional immunoprecipitation methods [1,9]. To overcome these limitations, a technique was developed for isolating chromatin-bound MSL complex that would allow for the identification of interacting factors by mass spectrometry [1,10]. Through the use of this chromatin immunoprecipitation followed by mass spectrometry (ChIP-MS) technique, MLE and JIL-1 kinase were both isolated as MSL complex-interacting factors. Importantly, this method identified CG1832 (CLAMP) as one of the top interactors.

In addition to its role in male-specific dosage compensation, we have recently demonstrated that CLAMP is an essential protein that localizes genome-wide, is required for the viability of both males and females, and plays a role in regulating chromatin accessibility across the genome [6,7,11]. To provide further insight into the essential function of the CLAMP protein, we identified interacting factors by performing immunoprecipitation for CLAMP under non-crosslinked and cross-linked conditions followed by mass spectrometry in male (S2) and female (Kc) cell lines. While our mass spectrometry approach did not identify any of the MSL complex components, an interaction between CLAMP and MLE has been previously reported [12].

Interestingly, we identified a new association between CLAMP and the Negative Elongation Factor (NELF) complex that is present in both male and female cell lines. Furthermore, we determined that CLAMP negatively regulates NELF recruitment to several highly paused target genes, which is the opposite function previously demonstrated for the similar GAGA Factor (GAF) protein that recognizes the same GA-rich *cis*-elements as CLAMP [13]. Therefore, the association between CLAMP and NELF has functional consequences at several promoters with paused RNA Polymerase II (RNA Pol II), including *hsp70*. Importantly, the novel set of factors associated with CLAMP that we identified provides a new resource for future experimentation on the diverse roles CLAMP plays at its thousands of binding sites throughout the genome.

## Results

### Immunoprecipitation of CLAMP after cross-linking identifies many putative interactors

To identify factors that associate with CLAMP *in vivo*, we performed two biological replicates of CLAMP immunoprecipitation under non-crosslinked conditions followed by mass spectrometry analysis from whole cell extracts of male (S2) and female (Kc) cells. As a complementary approach, we performed two biological replicates of ChIP followed by solution mass spectrometry from extracted nuclei of S2 and Kc cells (ChIP-MS). However, peptides were detected in only one of the two ChIP-MS S2 cell replicates and no peptides were detected in the Kc samples. To identify abundant non-specific proteins, we performed negative control immunoprecipitations using IgG in both Kc and S2 cells.

**Fig 1:**
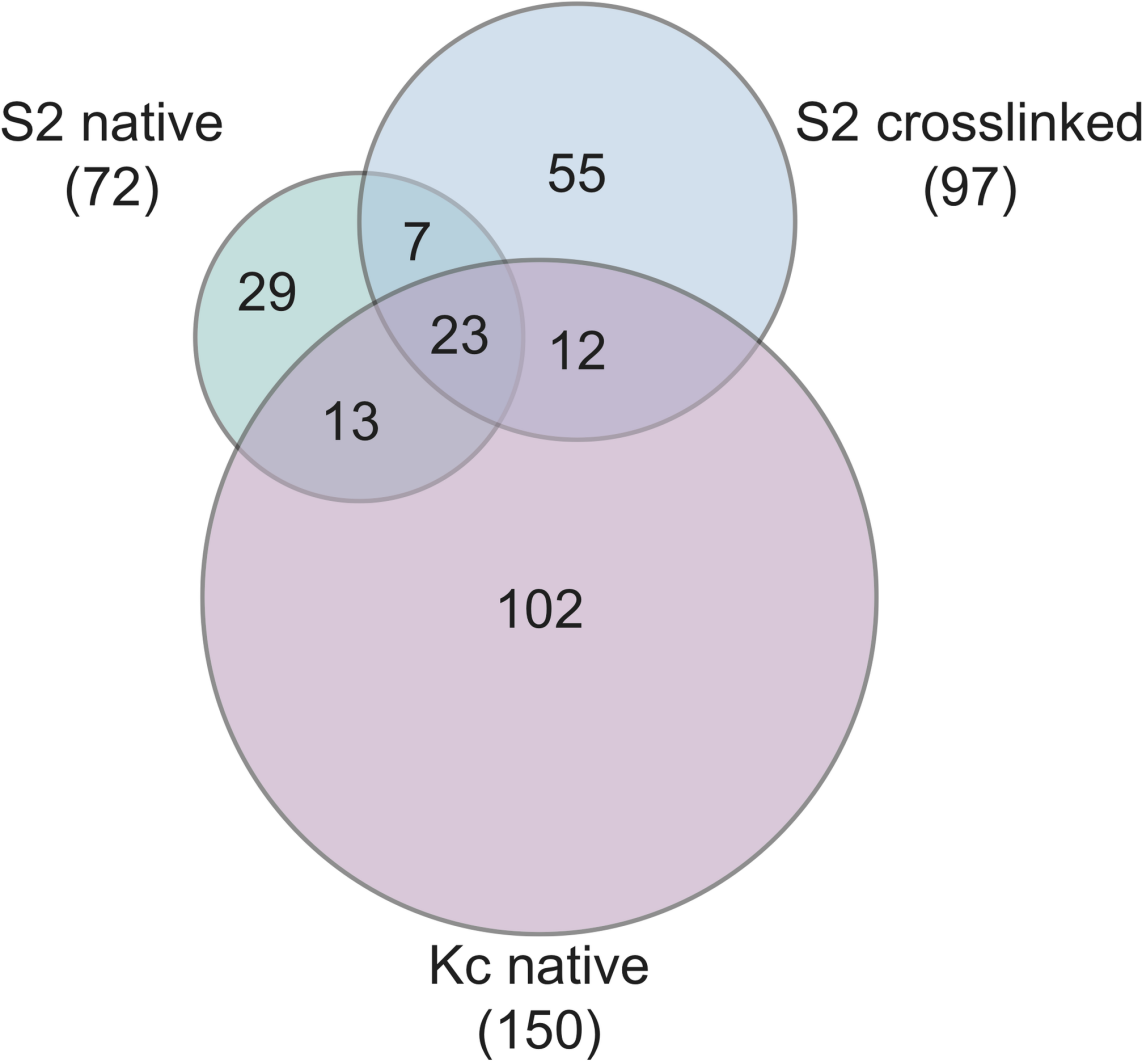
Comparison of proteins identified from mass spectrometry. The number of proteins identified from mass spectrometry in Kc and S2 cells under non-crosslinked conditions and S2 cells prepared under cross-linking conditions were compared to generate a Venn diagram. The cross-linking mass spectrometry approach identified 42 proteins previously identified under non-crosslinked conditions, and 55 previously unidentified factors. There are 23 proteins that were identified as CLAMP-interacting proteins using all three methods.

From our CLAMP immunoprecipitation without crosslinking samples, 72 proteins were identified as CLAMP interactors in S2 cells and 150 were identified in Kc cells (Fig 1). Of these, 36 were identified as associated with CLAMP in both S2 and Kc cell types (Fig 1). Next, we compared the results of the cross-linking approach to those obtained from the S2 and Kc non-crosslinking mass spectrometry technique (Fig 1, Table 1). In total, 97 proteins were identified using the ChIP-MS approach, 55 of which are absent from our S2 and Kc non-crosslinked mass spectrometry datasets (Fig 1, S2 Table). Of the 42 proteins found using both approaches, 23 (54.8%) were found in both S2 and Kc cells using the non-crosslinked approach (Fig 1, Table 1). Consequently, 64% (23/36) of interactors shared between S2 and Kc cells were also identified in S2 cells using the cross-linking method, suggesting that these interactions occur on chromatin (Fig 1, Table 1). Several of the proteins identified in all conditions are commonly identified in *Drosophila* mass spectrometry experiments investigating chromatin-bound proteins, such as Hsp70, Elongation Factor 1 and Ubiquitin (Table 1) [14,15]. It is likely that these factors do not functionally interact with CLAMP, although additional experiments are required to rule out this possibility.

**Table 1:** List of proteins identified in all three mass spectrometry approaches.

Protein Name
CLAMP*
Heat shock 70 kDA protein Cognate 5
Stress-sensitive B*
Elongation Factor 1 *
Negative Elongation Factor A
Ubiquitin-63E*
Heat shock 70 kDa protein cognate 4
Polyadenylate-binding protein
Ubiquitin-5E
Syncrip*
Clueless
Histone H2B
Histone H2A.v

While 23 different proteins were identified in all three mass spectrometry approaches, several had multiple isoforms. After combining proteins with more than one isoform, a total of 13 unique proteins were identified. The asterisk indicates proteins for which more than one isoform was identified.

We next determined which factors were identified in a cell-type specific manner. Of the proteins identified in only one cell type, 102 were found only in Kc cells (Fig 1A, S1 Table), 29 were identified only in S2 cells (Fig 1, S2 Table), and 55 were identified using the cross-linking method in S2 cells (Fig 1, S2 Table). Further comparison of the overlap between proteins identified in multiple samples indicates that 7 proteins are present in both S2 cell conditions but not in Kc cells (Fig 1, S3 Table). There are 12 proteins shared between Kc cells and the cross-linked S2 cells, whereas 13 proteins were found in Kc and S2 cells prepared under non-crosslinked conditions (Fig 1, S3 Table). Despite finding interactions present in one cell type and not the other, follow up studies will be necessary to determine if these differences simply arise because each dataset has not been sampled to saturation. Using a combination of non-crosslinked and cross-linking sample preparation, we were able to identify many factors that interact with CLAMP. Importantly, the majority (64%) of interactors identified using the non-crosslinked method were also identified using the cross-linking technique, suggesting that these factors likely frequently associate with CLAMP.

In both males and females, CLAMP binds to thousands of sites throughout the genome that are distributed among diverse genomic features, including transcription start sites (TSS), gene bodies, and enhancers [6,16]. This suggests that CLAMP may have many different functions. Consistent with the diverse occupancy patterns of CLAMP, both mass spectrometry approaches identified proteins with varying functions related to gene regulation including regulating RNA Pol II function, chromatin remodeling, and alternative splicing (Table 1, S1, S2 and S3 Tables). Several CLAMP-associated factors function as RNA binding proteins, including Alan Shepard (S2 Table) and Modulo (S3 Table), both of which have roles in epigenetic regulation of genome organization [17–19]. Interestingly, two CLAMP associated proteins, Squid (S1 Table) and Syncrip (Table 1) were previously known to interact with each other [20], suggesting that CLAMP may interact with these proteins as a complex.

### CLAMP associates with two subunits of the NELF complex

Previous work from our laboratory determined that CLAMP is an essential protein in males and females with a role in regulating global chromatin accessibility and transcription at TSS [6,7,11]. Therefore, we were most interested in CLAMP-associated factors with known roles at the TSS that were identified in all three sets of samples: Kc cells and S2 cells (both non-crosslinked and crosslinked conditions). One such factor is the transcriptional regulator Negative Elongation Factor A (NELF-A), which was identified as a top CLAMP interactor in all replicates of Kc and S2 cell immunoprecipitations as well as the ChIP-MS approach (Table 1). It is likely that the interaction between CLAMP and NELF-A is chromatin-associated because the ChIP-MS approach is performed on solubilized chromatin. NELF-A is a glutamine-rich subunit of the Negative Elongation Factor Complex that regulates promoter-proximal pausing of RNA Pol II and chromatin accessibility around TSS [21,22]. We previously reported that CLAMP regulates accessibility upstream of transcription start sites genome-wide [11]. Therefore, the association between CLAMP and NELF suggests that CLAMP-mediated accessibility at TSS may occur through regulation of NELF recruitment.

Due to the large size of NELF-A (~135kDA), there is a possibility that the identification of this protein in all samples is simply due to the fact that its peptides are likely to be detected frequently. To control for differences in the length of proteins, we tested whether the interaction between NELF and CLAMP was enriched in the immunoprecipitation with the anti-CLAMP antibody compared to the IgG control after normalizing for protein length (normalizing for molecular weight or number of peptides yielded comparable results). We calculated an enrichment score for the proteins that were identified in all three samples (S2 non-crosslinked, Kc non-crosslinked, and S2 cross-linked). First, we normalized the number of uniquely identified peptides to the length of the protein. Next, the length-normalized number of peptides identified in the negative IgG control was subtracted from the CLAMP immunoprecipitation to generate enrichment compared to IgG. After normalization, any protein with a negative enrichment value (enrichment score ≤ 0) in two or more of the samples was removed from the list, leaving 26 total proteins and a list of 13 after isoforms were combined (S4 Table). When normalizing peptide counts in this way, we were unable to discount NELF-A from the list of interactions for both techniques. Moreover, identifying an association between CLAMP and NELF-A with the cross-linking technique indicates that this interaction is likely to occur frequently and on chromatin.

**Fig 2:**
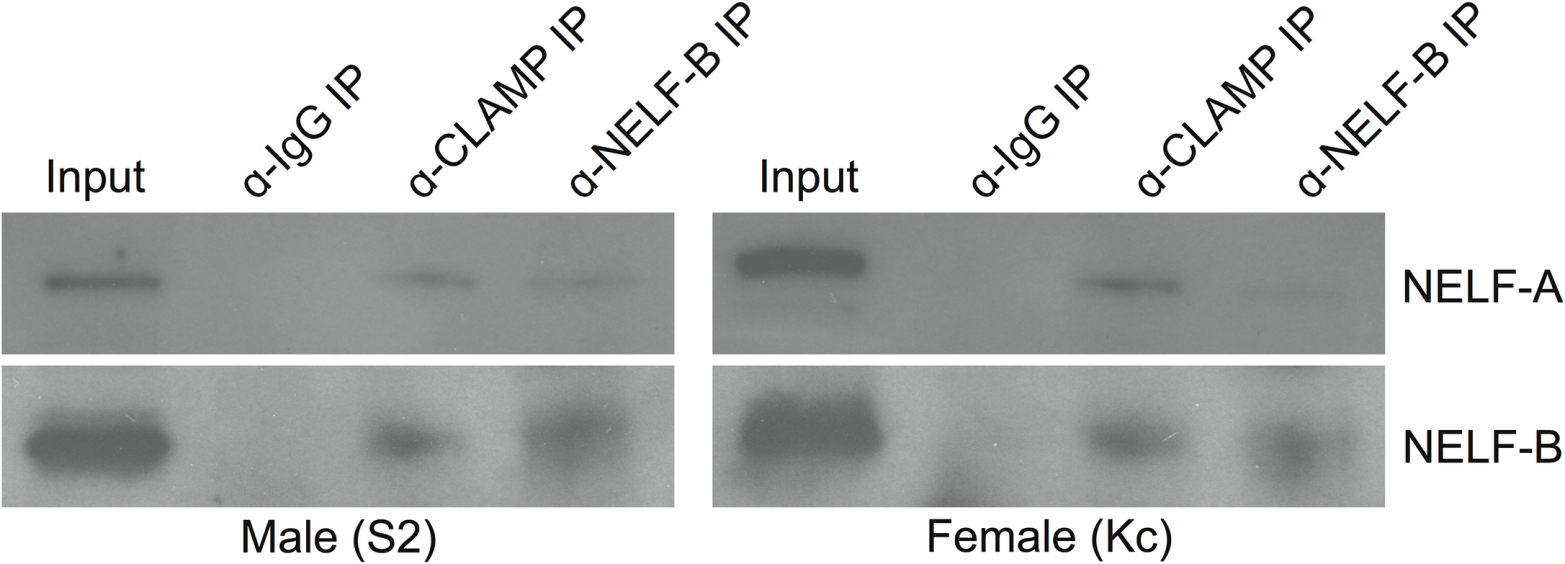
CLAMP interacts with the NELF-A and NELF-B subunits of the NELF complex. Immunoprecipitation of CLAMP or NELF-B was performed and samples were immuno-blotted for the NELF-A (top row) and NELF-B (bottom row) subunits of the NELF complex. CLAMP associates with both NELF subunits in male (S2) and female (Kc) cells, indicating that it likely interacts with the entire NELF complex.

Our goal was to validate the interaction between CLAMP and the NELF complex, however our mass spectrometry results only identified the NELF-A subunit of the NELF complex and not other components (Table 1). Since all components of the NELF complex are necessary for its function [23], we reasoned that the absence of other NELF components, such as NELF-B, in our mass spectrometry results might be due to a lack of sampling saturation. To address this, we performed an immunoprecipitation for CLAMP followed by western blotting for two subunits of the NELF complex: NELF-A and NELF-B. In addition to performing a CLAMP immunoprecipitation, we also performed a NELF-B immunoprecipitation to ask whether NELF-B co-immunoprecipitates CLAMP. By co-immunoprecipitation, we found that CLAMP associates with both the NELF-A and NELF-B subunits of the NELF complex (Fig 2). Based on these results, we conclude that the interaction between CLAMP and NELF is not specific to NELF-A, making it likely that CLAMP associates with the entire NELF complex.

### *In vivo* CLAMP, GAGA-factor, and NELF genomic binding sites overlap

**Fig 3:**
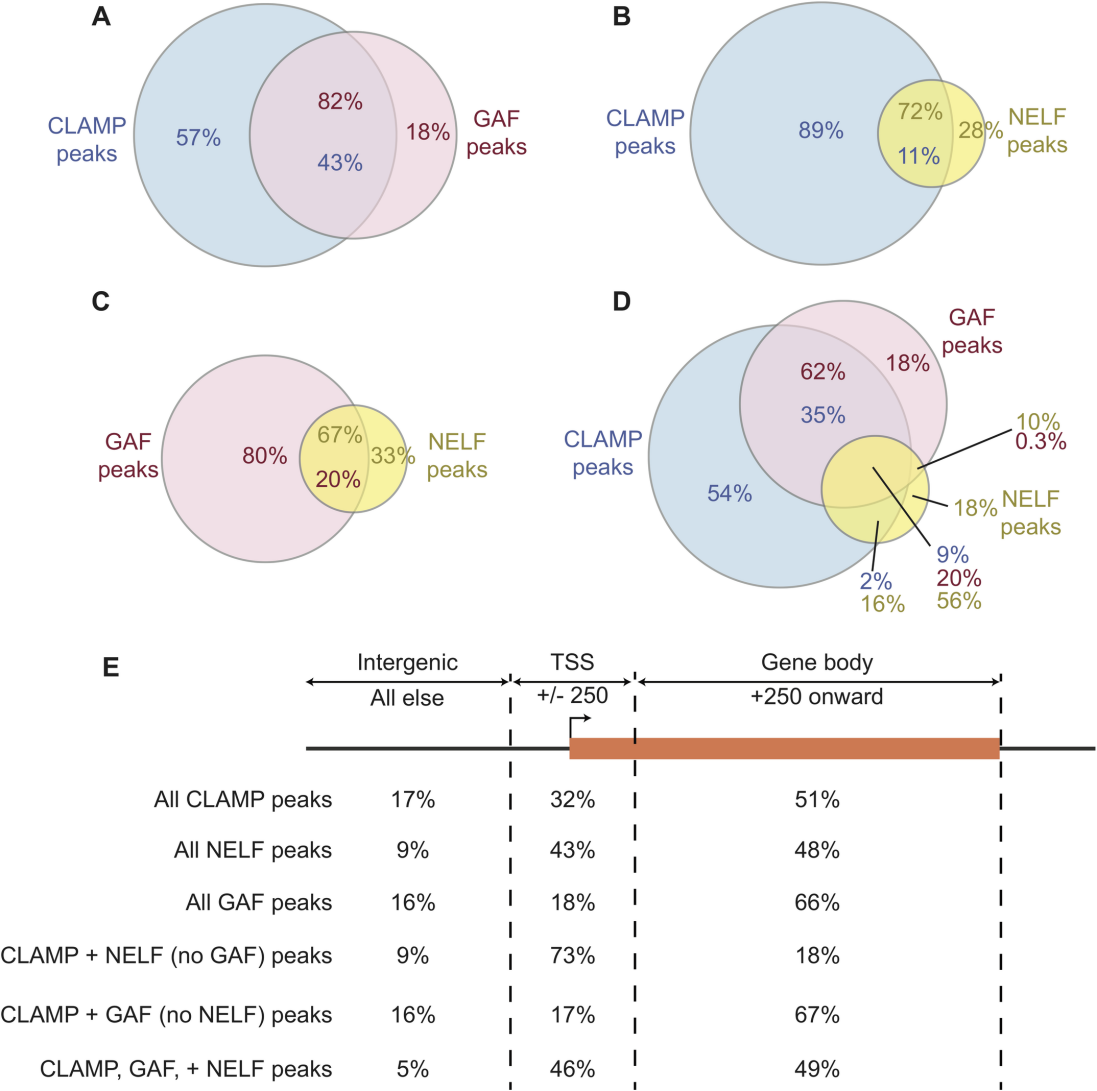
Overlap and distribution relative to genes of CLAMP, GAF, and NELF ChIP-seq occupancy. Comparison of CLAMP and GAF ChIP-seq peaks with NELF-B ChIP-chip peaks was performed to determine the overlap between factor occupancy. The numbers in dark green represent the percentage of CLAMP peaks, dark red are the percentage of GAF peaks, and dark blue are the percentage of NELF peaks. (A) Only 43% of CLAMP peaks overlap with GAF, whereas the majority (82%) of GAF peaks overlap with CLAMP. (B) A small fraction (11%) of CLAMP peaks overlap with NELF, while most NELF peaks also contain CLAMP (72%). (C) Fewer NELF peaks (67%) associate with GAF than with CLAMP (72%). (D) Venn diagram describes the percentage of CLAMP, GAF, and NELF peaks that overlap with each of the other factors. (E) CLAMP, GAF, and NELF peaks were categorized as either within +/- 250bp centered on the transcription start site (TSS), between +250bp and the end of the gene (gene body), or intergenic (all other peaks). The percentages of total CLAMP, GAF, or NELF peaks that fall within each of these regions are in the first three rows. Next, the distribution of CLAMP peaks that are also occupied by NELF, GAF, or both is shown in the last three rows.

We were interested in further defining the interaction between CLAMP and NELF by determining how their *in vivo* binding sites overlap using published CLAMP ChIP-seq [6] and NELF ChIP-chip data [24]. Overlapping binding sites do not necessarily indicate that these factors co-occupy these sites concurrently since occupancy measurements were obtained from ChIP-seq/chip experiments that involve cross-linking a population of cells. However, overlapping peaks suggest these factors may have a functional relationship with each other and would support our mass spectrometry results that suggest these factors interact on chromatin. For comparison, we also looked at the overlap of CLAMP and NELF with GAGA-factor (GAF) binding sites [25]. All of the data sets we analyzed were derived from the same (*Drosophila* S2) cell type that was used for the modENCODE project [6,24,25]. GAF is a well-studied GA-repeat binding transcription factor that recruits NELF to TSS to regulate pausing of RNA Pol II [13,14]. CLAMP and GAF recognize similar GA-rich motifs and both have zinc finger and glutamine-rich domains [6,26]. Consistent with CLAMP and GAF having the same binding motif, 43% of CLAMP peaks overlap with 82% of GAF peaks (Fig 3A). Therefore, most *in vivo* GAF binding sites are also CLAMP binding sites but fewer CLAMP binding sites are also GAF binding sites (Fig 3A). More than 81% of the CLAMP sites that overlap GAF sites (35% of CLAMP peaks) overlap with only GAF and not NELF (Fig 3D). Similar to GAF, NELF is not present at the majority of CLAMP peaks (89%), but CLAMP is present at most NELF peaks (72%) (Fig 3B). Approximately half of the CLAMP peaks have at least one of the other two factors associated with it, whereas all three factors overlap at only 9% of CLAMP sites (Fig 3D). As a result, both GAF and NELF are more likely to have at least one other factor associated with a binding site than CLAMP, which has more unique sites (54% CLAMP sites vs. 18% unique sites for both GAF and NELF) (Fig 3D). The majority of NELF peaks (67%) also overlap with GAF peaks (Fig 3C), and more than half of all NELF sites (56%) overlap with both CLAMP and GAF (Fig 3D). The high percentage of NELF peaks that contain GAF, CLAMP or both GA-binding factors is consistent with the lack of sequence-specific binding factors in the NELF complex [27] and the requirement for a GA-repeat sequence for NELF recruitment [28].

We next determined the distribution of CLAMP, GAF and NELF peaks to examine whether there is a specific location relative to genes that is enriched for overlaps between peak sets. We found that half of CLAMP peaks (51%) and the majority of the GAF peaks (66%) are located within gene bodies (Fig 3E, S5 Table). NELF peaks are distributed almost evenly between transcription start sites and gene bodies (Fig 3E, S5 Table). We also analyzed the genic location of CLAMP peaks that overlap with NELF, GAF, or both. Similar analyses were performed for GAF and NELF peaks (S5 Table). Of the CLAMP peaks that are also enriched for NELF, but not GAF, most are located at TSS (73%) (Fig 3E, S5 Table), which is consistent with the well-established roles for NELF in transcriptional pausing at promoters and release of RNA Pol II into gene bodies [24]. In contrast, most (67%) of the sites shared by CLAMP and GAF are present within gene bodies (Fig 3E, S5 Table). The majority of sites shared by all three factors are almost evenly distributed between gene bodies (49%) and transcription start sites (46%). Within intergenic regions, there is a larger proportion of sites where CLAMP and GAF overlap (16%) than is occupied by CLAMP and NELF (9%) or all three factors together (5%) (Fig 3E, S5 Table). Our identification of overlap between CLAMP and GAF occupancy at intergenic regions is consistent with a recent discovery that identified CLAMP and GAF within the same insulator complex called the Late Boundary Complex (LBC) [29], suggesting a role in insulator function for CLAMP and GAF within these regions.

### CLAMP negatively regulates NELF enrichment to highly paused genes

From our analysis of available genome-wide ChIP data sets, we were most interested in the observation that CLAMP and NELF occupancy overlap at TSS (Fig 3). Previous studies suggest that GAF promotes recruitment of NELF to transcription start sites because GAGA elements are located at a majority of NELF-regulated paused genes [30,31]. GAF recruitment is necessary for maintaining an open chromatin environment in these regions [13,24,25,28]. Recently, we have reported a role for CLAMP in promoting the positioning of chromatin accessibility at transcription start sites in both males and females [11]. Based on these data, we hypothesized that CLAMP functions similarly to GAF as a sequence-specific recruitment factor that modulates NELF occupancy.

To test our hypothesis, we measured how CLAMP regulates recruitment of NELF to promoters by performing three biological replicates of ChIP-qPCR for NELF-B after control (*gfp*) RNAi and *clamp* RNAi treatment in S2 cells. We measured NELF enrichment at genes that are bound by CLAMP within their upstream promoter region [6]. These promoters also exhibit changes in chromatin accessibility following CLAMP depletion as measured by a MNase-sequencing experiment [11]. Within this subset of CLAMP-regulated promoters, we chose promoters that were also NELF-bound [30] and exhibited differing degrees of RNA Pol II pausing [32] to evaluate whether the pausing status of the gene influenced the effect of CLAMP on NELF recruitment. The degree of pausing was previously defined using a metric called the Pausing Index (PI) [32], which is the ratio of RNA Pol II ChIP-seq enrichment within the promoter region (+/- 250 bp centered on the transcription start site) compared to the gene body (+500 bp from the TSS to gene end).

**Fig 4:**
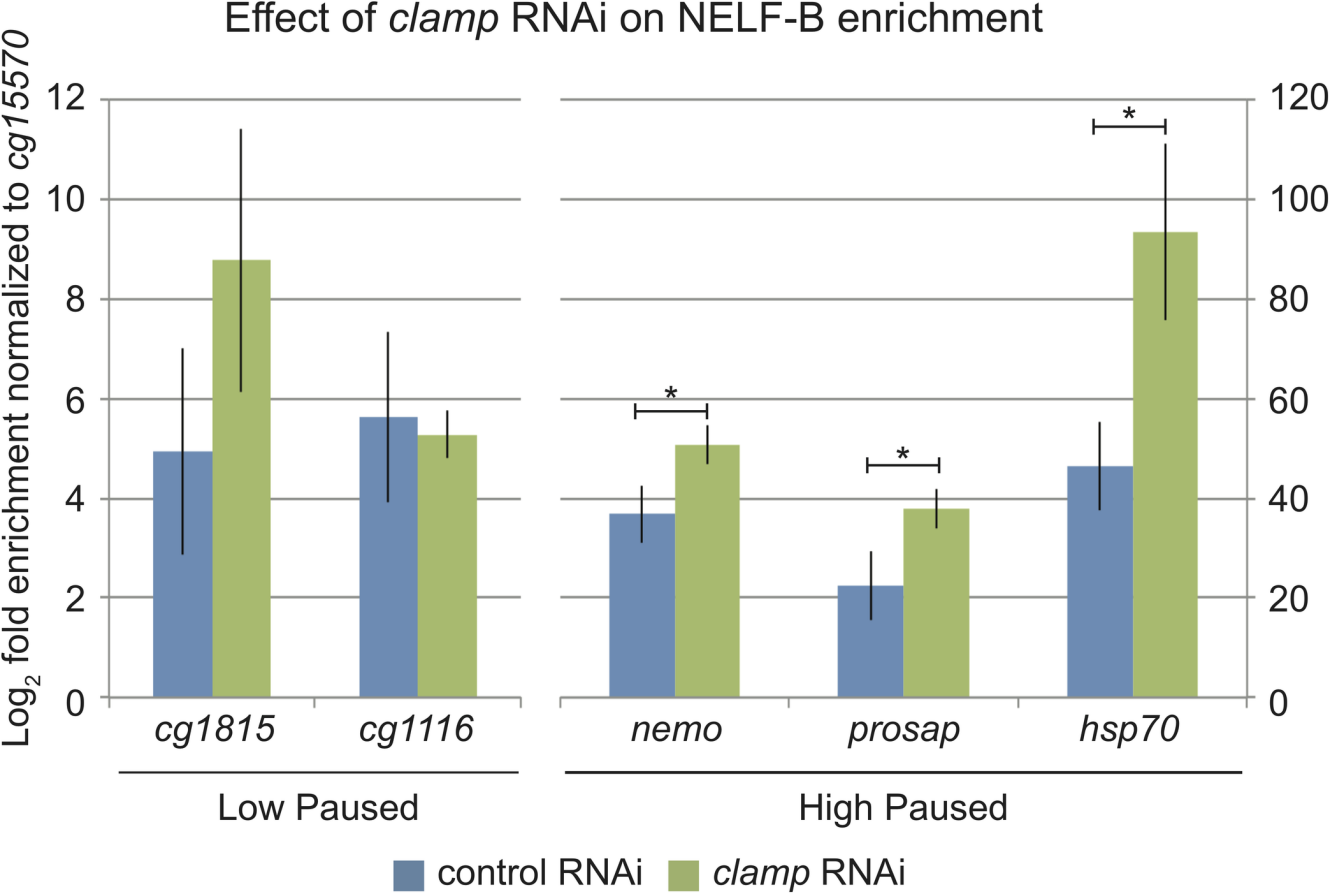
CLAMP inhibits NELF recruitment to highly paused genes. Chromatin immunoprecipitation of NELF-B was performed from S2 cells treated with either *gfp* control (blue) or *clamp* (green) RNAi. The values for log_2_-fold enrichment over Input are shown after normalizing internally to a control locus (*cg15570*) that is unbound for CLAMP or NELF-B. These values were then normalized to Input to generate the log_2_-fold enrichment value. Three separate biological replicates were averaged and the standard error of the mean was calculated (error bars are +/- 1 S.E.M.). Significance was determined using Kruskal-Wallis test by ranks, where the asterisk indicates a p-value <0.05. The y-axis on the left shows enrichment scores for the low paused genes, while the y-axis on the right indicates the values for the high paused genes. The highly paused genes have greater enrichment of NELF-B than the lowly paused genes, as expected.

To determine whether CLAMP regulates NELF recruitment, we performed NELF ChIP-qPCR at two genes with a low PI (PI<1) and three genes with a high PI (PI>1). At the two promoters with low PI, there was no effect on NELF enrichment following *clamp* RNAi treatment (Fig 4). However, at the three promoters with high PI, including the well-studied *hsp70* promoter, we observed an increase in the enrichment of NELF following *clamp* RNAi treatment (Fig 4). In addition to CLAMP and NELF, GAF is also enriched at these promoters as determined by ChIP-seq profiles [25]. It has been previously demonstrated that GAF is required to recruit NELF to the *hsp70* promoter [13]. These results suggest that CLAMP may function antagonistically to GAF to modulate NELF recruitment levels, specifically at genes with high levels of RNA Pol II pausing such as *hsp70*. In the future, it will be critical to define how the relationship between CLAMP and GAF influences NELF enrichment genome-wide.

**Fig 5:**
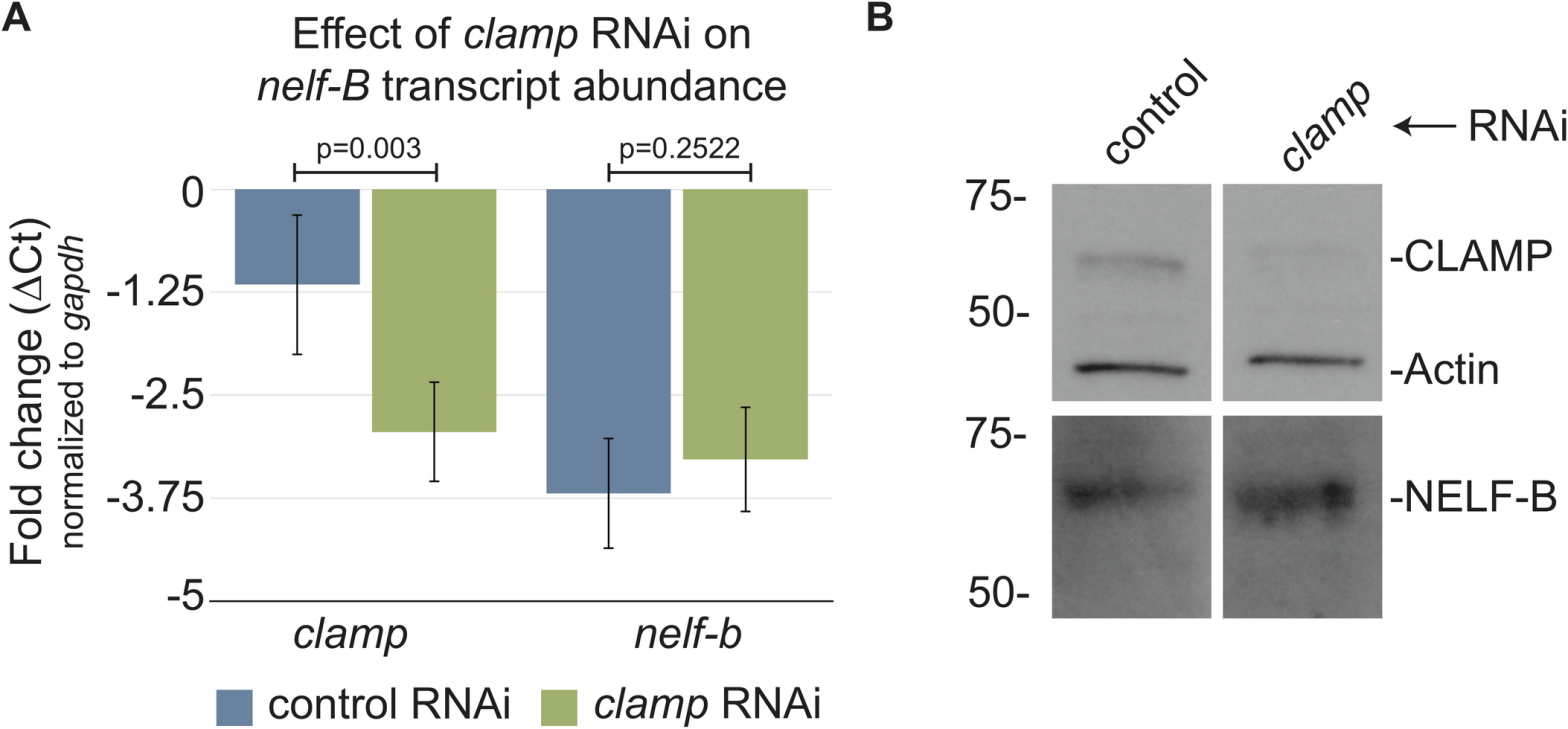
CLAMP does not regulate NELF-B protein abundance. (A) Transcript abundance for *clamp* and *nelf-b* was measured to determine the difference in transcript abundance between control (*gfp*, blue) and *clamp* (green) RNAi treatment by qPCR. The average fold change (ΔCt compared to *gapdh*) from four biological replicates is shown for both *clamp* and *nelf-b* transcripts. As expected, abundance of *clamp* is reduced after *clamp* RNAi, while *nelf-b* transcript abundance is not affected compared to the control RNAi. The error bars represent +/- 1 S.D., with p-values indicated. (B) Protein accumulation was measured by western blot for both CLAMP and NELF-B after of *clamp* and *gfp* control RNAi. RNAi targeting *clamp* reduces the amount of CLAMP protein but has no effect on NELF-B protein levels. Actin is used as a loading control.

CLAMP localizes to TSS throughout the genome and regulates the expression of thousands of genes [6,11], raising the possibility that the effects on NELF complex occupancy seen by ChIP after *clamp* RNAi were due to CLAMP regulating expression of NELF complex components. To address this possibility, we tested whether *clamp* RNAi alters *nelf-b* mRNA and protein abundance. We tested the expression of NELF-B because this subunit is a structural component of the complex and all four subunits are required for complex function [23]. We found that while *clamp* RNAi significantly reduces the amount of *clamp* transcript, there was no effect of *clamp* RNAi on transcript abundance of *nelf-b* (Fig 5A). Furthermore, we tested NELF-B protein abundance and determined that *clamp* RNAi does not alter NELF-B protein levels (Fig 5B). Therefore, we conclude that changes in NELF enrichment at highly paused genes after *clamp* RNAi treatment are due to changes in NELF occupancy and not protein levels.

Overall, we have identified a non-sex-specific association between CLAMP and NELF that reduces NELF occupancy at several paused promoters. It is known that GAF positively regulates NELF recruitment to the *hsp70* promoter [13], where we have now shown that CLAMP negatively influences NELF occupancy. Therefore, it is possible that differential occupancy of CLAMP and GAF regulates NELF recruitment to assure RNA Pol II pausing is tightly controlled.

## Discussion

Understanding the physical associations between transcriptional regulators on chromatin is essential to revealing the mechanisms by which genes are regulated. While traditional methods of immunoprecipitation followed by mass spectrometry are powerful, chromatin-associated interactions are more challenging to identify [33]. ChIP-MS methodologies provide the means to identify these interactions. By using a combination of non-crosslinked and cross-linking approaches, we identified numerous factors that associate with the essential CLAMP transcription factor. Importantly, many proteins found using the non-crosslinked approach were also found by cross-linking, providing support that these interactions occur on chromatin. This indicates that our MS datasets may be comprised of factors that frequently or stably interact with CLAMP. Whereas other important factors that associate relatively infrequently with CLAMP for context-specific functions at different genomic locations have not yet been identified. Moreover, tissue-specific proteomic analyses are required to fully understand the extent of the CLAMP interactome *in vivo*.

Using ChIP-MS, we identified proteins with diverse roles in regulation of gene expression, such as RNA Pol II pausing (e.g. NELF-A), insulator function (e.g. Alan Shepard), alternative splicing, and mRNA localization (e.g. Syncrip). Could CLAMP mediate the interaction between these proteins and chromatin to promote context-specific functions at different genomic locations? It is possible that CLAMP functions as an adapter protein that mediates many different protein-DNA interactions, because CLAMP is composed of a glutamine-rich domain and a DNA binding zinc finger domain. For example, on the male X-chromosome, CLAMP promotes recruitment of MSL complex [6], while at promoters there may be competition between GAF and CLAMP for NELF recruitment. It would then follow that CLAMP may promote diverse functions through the recruitment of specific factors to distinct locations within the genome. Understanding these diverse roles will require a comprehensive view of all interactions that CLAMP mediates. It is likely that our dataset is an underestimation of the complete CLAMP interactome, as it is enriched for large and highly abundant proteins. Nevertheless, our mass spectrometry results provide candidates for beginning to understand the diverse context-specific functions for CLAMP, which associates with thousands of genomic loci throughout the genome including promoters, gene bodies, and enhancers [6,7,11,29,34].

Previous work from our laboratory determined that CLAMP regulates chromatin accessibility at promoters genome-wide in both males and females [11]. These changes in chromatin accessibility occurred independently of histones, because ChIP of the core Histone 3 (H3) protein indicated no differences in histone occupancy following *clamp* RNAi treatment. We hypothesized that other non-histone factors may be responsible for the observed changes in accessibility. From our mass spectrometry datasets, we identified the transcriptional regulator NELF, which has known roles in promoting an open chromatin environment within the nucleosome-depleted upstream promoter region [22,24]. Indeed, ChIP-qPCR following *clamp* RNAi treatment indicates that CLAMP negatively regulates NELF occupancy at the genes tested that have high pausing indices. This is the opposite function of the known positive relationship between the similar GAF protein and NELF [13,24,25,28]. Therefore, it is possible that an antagonistic relationship exists between CLAMP and GAF for recruitment of NELF, such that their competition precisely regulates NELF occupancy at promoters. Competition between CLAMP and GAF may be necessary to fine-tune regulation of RNA Pol II release into productive elongation which would ensure precise regulation of transcript levels.

In addition to NELF-A, we identified several CLAMP-interacting factors that function as RNA binding proteins, including Alan Shepard (Shep) and Modulo (mod) (S2 and S3 Tables). Shep interacts with the gypsy insulator complex, and is a negative regulator of gypsy element insulator activities, specifically in the central nervous system [17]. The gypsy element is an insulator sequence that exhibits enhancer-blocking activity. When associated with insulator protein complexes, insulator sequences contribute to the overall structure of insulator bodies, which are located at the periphery of the nucleus to form the boundaries between topologically associated domains [35]. Interestingly, we have recently discovered an association between CLAMP and another insulator complex called the Late Binding Complex (LBC) present only in the late embryo that functions with GAF at the *Fab-7* insulator [29]. Therefore, it is possible that CLAMP has a role in regulating insulator complex function.

In addition to Shep, the ChIP-MS approach identified the RNA-binding protein Modulo as a novel CLAMP interactor present in both Kc and S2 cell samples (S3 Table). Mod has been classically studied for its role as a suppressor of position effect variegation (PEV) [18]. PEV is a phenomenon where a gene that is experimentally placed in proximity to constitutive heterochromatin is randomly expressed or repressed in a mosaic nature due to the fluidity of the heterochromatic border. Mod has been identified as a suppressor of variegation [Su(var)], suggesting that it participates in the formation of heterochromatin. In addition to having a role in regulating heterochromatin formation, Mod has an additional role as an RNA binding protein when associated with the nucleolus [19]. It is possible that CLAMP may associate with Mod when it is performing either one or both of these specific functions, an intriguing possibility for future exploration.

Interestingly, two CLAMP associated proteins, Squid (Sqd) and Syncrip (Syp) were previously known to interact with each other. It is therefore possible that CLAMP interacts with these two proteins as a protein complex. Sqd was identified in only female Kc cells, consistent with its primary function in the ovary [36], and Syp was identified in all three conditions, consistent with its roles in multiple tissues (Table 1 and S1 Table) [37,38]. Sqd and Syp are both members of the heterogeneous nuclear ribonucleoprotein (hnRNPs) class of proteins, which have functions in regulating RNA processing, localization, and alternative splicing [20,39]. Together, Sqd and Syp have an essential role in mRNA localization that occurs during early axis specification of the *Drosophila* oocyte and embryo [20]. Specifically, Sqd and Syp associate with each other to regulate cellular localization of the *oskar* and *gurken* transcripts in the oocyte [20]. CLAMP is strongly enriched in the developing oocyte and early embryo [40], making it possible that it associates with Sqd and Syp during this developmental time point. It is important to note that an interaction between these three proteins would require additional confirmation because our data set was generated from a cell line and not a tissue or whole embryo. Further *in vivo* studies investigating this interaction will allow us to determine if there are functional consequences for the interaction between CLAMP and Sqd/Syp involving processing of *oskar* and *gurken*.

Overall, the essential CLAMP protein interacts with a diverse pool of proteins and it is likely that differential interactions across the genome precisely modulate gene expression and chromatin organization. Future analysis of the functional and physical relationships between CLAMP and our newly identified associated proteins will elucidate the many context-specific roles for the essential CLAMP protein.

## Materials and methods

### Cell culture conditions

*Drosophila* S2 and Kc167 modENCODE cell lines from the *Drosophila* RNAi Screening Center were maintained at 25°C in Schneider’s media (ThermoFisher Scientific) supplemented with 10% Fetal Bovine Serum and 3.5% Antibiotic-Antimyotic (ThermoFisher Scientific). Cells were passaged every 2–3 days to maintain an appropriate cell density.

### Immunoprecipitation and mass spectrometry of CLAMP without crosslinking

#### Preparation of S2 and Kc cell protein lysate

Male (S2) and female (Kc) *Drosophila* tissue culture cells were grown to a cell concentration of 7x10^6^ cells/mL in T225 tissue culture flasks. Cells were harvested from the flask by scraping and centrifuged for 5 minutes at 2,500 rpm at 4°C. In total, two biological replicates per cell type were collected. The supernatant was removed and cell pellets were washed twice in 5mL of cold PBS. The washed cell pellets were then resuspended in 5X volume of Buffer A (10mM HEPES pH 7.9, 1.5mM MgCl_2_, 10mM KCl, 0.5mM DTT, 1X protease inhibitors). Cells were incubated on ice for 15 minutes before dounce homogenization with an A pestle. The cytoplasmic fraction was collected after centrifugation at 4°C for 20 minutes at 700xg. The remaining nuclear pellet was resuspended in 3 times volume in Buffer B (20mM HEPES pH 7.9, 20% Glycerol, 0.5% NP-40, 200mM KCl, 0.5mM EDTA, 1mM EGTA, 1X protease inhibitors). Following resuspension, nuclei were dounce homogenized with a B pestle. The nuclear debris was then pelleted by centrifugation at 10,000xg for 10 minutes at 4°C. 1mL aliquots of the cytoplasmic and nuclear fractions were prepared in 1.5mL Protein LoBind Eppendorf tubes (Eppendorf) and flash frozen in liquid nitrogen for storage at -80°C.

#### Immunoprecipitation of CLAMP and IgG

Magnetic anti-CLAMP beads were prepared to a final concentration of 10mg/mL by coupling rabbit anti-CLAMP antibody (SDIX) to magnetic beads according to the instructions provided with the Dynabeads Antibody coupling kit (ThermoFisher Scientific). Both prepared anti-CLAMP and purchased anti-IgG (anti-rabbit IgG M-280 Dynabeads) were blocked to reduce background the night prior to the immunoprecipitation. First, the beads were washed 3 times for 5 minutes in 500uL Tris-NaCl Wash (50mM Tris, 500mM NaCl, 0.1% NP-40) by rotating at 4°C. The beads were next suspended in block buffer (3.3mg/mL of yeast tRNA extract prepared in 20mM HEPES, pH7.9, 20% Glycerol, 0.5% NP-40, 200mM KCl, 1mM EDTA, and 2mM EGTA) and rotated overnight at 4°C. The next day, beads were washed 3 times for 5 minutes in block buffer without yeast tRNA by rotating at 4°C. After the final wash, beads were resuspended in the same amount of block buffer as the starting volume.

To 1mL of previously prepared nuclear extract, 100μL of blocked anti-CLAMP or anti-IgG magnetic Dynabeads were added. The nuclear extracts and beads were then rotated for 1 hour at 4°C. Afterward, the beads were collected and supernatant discarded. The beads were then washed three times in Tris-NaCl wash (50mM Tris, 500mM NaCl, 0.1% NP-40) by rotating for 5 minutes at 4°C and clearing by using a magnetic rack. To elute proteins from the beads, 100μL of 1% SDS was added and the beads were boiled for 10 minutes at 95°C. To the eluate, 300μL of ultra pure water was added and the tubes gently vortexed. After collecting the beads on a magnetic rack, the eluate was saved in a fresh Protein LoBind Eppendorf tube.

#### Protein clean up, trypsin digest, and peptide desalting

500μg of total protein in a volume of 100uL was prepared for cleanup following the manufacturer’s protocol for the ReadyPrep 2-D cleanup (BioRad). The cleaned proteins were resuspended in a buffer containing 100mM Tris and 6M Urea to obtain a concentration of 10μg/μL. The samples were reduced by adding 2.5 μL of 200mM DTT and incubated at room temperature for 1 hour. Next, the samples were alkylated by adding 10μL of 200mM Iodoacetamine and incubating for 1 hour at room temperature in the dark. Finally, the reaction was neutralized with the addition of 10μL 200mM DTT and incubated for 1 hour at room temperature. The samples were then diluted with 400μL of ultra pure water to perform a trypsin digest overnight at 37°C. Trypsin was added at a ratio of 1mg trypsin for every 20mg of protein sample.

After trypsin digestion, the peptides were concentrated by drying in a speed vacuum to approximately 20μL. Peptides were then desalted following the manufacturer’s protocol for ZipTips with a C18 resin (Millipore).

### Immunoprecipitation and mass spectrometry of CLAMP under cross-linking conditions

#### Isolation and cross-linked of nuclei

Two biological replicates containing approximately 1x10^8^ *Drosophila* S2 cells each were collected by scraping a T225 flask followed by centrifugation for 3 minutes at 2,000xg at 4°C. The pelleted cells were washed once in 10mL of cold PBS and a small aliquot was taken to obtain cell count. Next, the cells were resuspended in 1mL ice cold Buffer A before adding additional Buffer A (10mM HEPES pH 7.9, 10mM KCl, 1.5mM MgCl_2_, 10% Glycerol, 340mM Sucrose, 1X protease inhibitors, and 1mM DTT) to achieve a cell concentration of ~1x10^7^ cells/mL. To the suspended cells, 100μL of 10% TritonX-100 was added and mixed gently. The mixture was transferred to a clean dounce homogenizer, and incubated for 10 minutes on ice. The cells were gently homogenized for 15 strokes using an A pestle. The disrupted cells were then transferred to a 50mL conical tube to pellet the nuclei by centrifugation at 500xg for 5 minutes at 4°C.

Excess cytoplasm was removed by first resuspending the nuclei with a wide bore pipette tip in 1mL of Buffer A+T (Buffer A + 0.1% TritonX-100). To the 1mL, an additional 9mLs of Buffer A+T was added. A small aliquot was taken at this point to count nuclei. Next, an additional 20mLs of Buffer A+T were added and the nuclei gently mixed. The supernatant was removed following centrifugation of the nuclei at 500g for 5 minutes at 4°C. The washed nuclei pellet was next resuspended in 1mL of ice cold Buffer A+T. To this, 11mLs of cross-linking solution (10mM HEPES pH 7.9, 10mM KCl, 1.5mM MgCl_2_, 10% Glycerol, 340mM Sucrose, 1% formaldehyde, 0.1% TritonX-100) was added and the nuclei were mixed end-over-end for 10 minutes at room temperature. The cross-linking reaction was quenched by adding 2.5M glycine to a final concentration of 125mM. Fixed nuclei were incubated for 5 minutes on ice before centrifugation at 500xg for 5 minutes at 4°C. The supernatant was discarded and the crosslinked nuclei were resuspended in 1mL of ice cold Buffer A+T.

#### Isolation of chromatin and digestion

To lyse the nuclei, they were first pelleted by centrifugation at 500xg for 5 minutes at 4°C. Next, the nuclei were resuspended in 1mL of Buffer B (3mM EDTA, 0.2mM EGTA, 0.1% TritonX-100, 1X protease inhibitors, 1mM DTT) before incubating on ice for 10 minutes. The lysed nuclei were then pelleted by centrifugation at 3,000xg for 3 minutes at 4°C and the soluble nucleoplasm removed. The insoluble chromatin was then washed by adding 1mL of Buffer B and incubating on ice for 10 minutes before centrifugation at 3,000xg for 3 minutes at 4°C. The washed chromatin was next resuspended in 570μL of Buffer B and 30μL of 10% TritonX-100 was added before incubating on ice for 5 minutes. The samples were split into 600μL fractions for sonication. Chromatin was sonicated using a water bath sonicator (Bioruptor, Diagenode) for 3 cycles of 5 minutes each, with the sonicator programed to pulse on for 30s, then off for 30s (50 bp-150 bp DNA length).

To the sonicated chromatin, 450μL of Buffer B and 50μL of 10% Triton-X 100 were added before incubation with rotation for 15 minutes at 4°C. The solubilized chromatin was then separated from the insoluble fraction by centrifugation at 3000xg for 3 minutes 4°C. A 950μL aliquot was taken for each immunoprecipitation.

For each immunoprecipitation, 27μL of 5M NaCl and 23μL of 1M Tris, pH 8.0 was added. To the prepared protein lysate, 2μL of either CLAMP (rabbit, SDIX) or IgG (rabbit, Millipore) antibody were added and the samples were incubated overnight with rotation at 4°C. Next, samples were incubated for 2 hours with 100μL Protein A sperm blocked agarose beads (Millipore). The unbound material was removed from the beads before washing. First beads were washed twice with 750μL 135mM ChIP Wash buffer (0.1%SDS, 1% TritonX-100, 2mM EDTA, 20mM Tris, pH 8.0, 135mM NaCl). For each wash the beads were incubated with rotation for 3mins at 4°C, followed by centrifugation at 1,000xg for 3 minutes. Next, the beads were washed once in 750μL of 200mM ChIP Wash Buffer (0.1%SDS, 1% TritonX-100, 2mM EDTA, 20mM Tris, pH 8.0, 200mM NaCl). Finally, the beads were eluted three times by added 300μL ChIP Elution buffer (100mM Sodium Bicarbonate, 1% SDS). For each elution, the beads were incubated at 50°C and shaken in a thermomixer set to 1200rpm for 10 minutes.

#### Mass spectrometry

Input, CLAMP, and IgG immunoprecipitated samples were prepared for mass spectrometry analysis as described above for the non-crosslinked samples with the exception that Pierce^TM^ C18 Spin Tips were used for desalting (ThermoFisher). Nano-LC−MS/MS Analysis: Tryptic peptides were fractionated on a 75 μm × 12 cm column containing 3 μm Monitor C18 resin (Orochem Technologies, Inc., Lombard, IL) and having an integrated 10 μm ESI emitter tip (“Self-Pack” PicoFrit column, New Objective, Woburn, MA). Solvent A was 0.1 M acetic acid in water and solvent B was 0.1 M acetic acid in acetonitrile. Peptides were eluted with a linear acetonitrile gradient (0−70% solvent B over 60 min), operated at 200 nL/min using an Agilent 1200 HPLC (Agilent Technologies, Santa Clara, CA) and passive split flow. The column eluate was introduced directly onto a LTQ Orbitrap Velos mass spectrometer (Thermo Scientific, San Jose, CA) with a 1.8 kV ESI voltage. Full MS scans in the m/z range of 300−1700 at a nominal resolution of 60,000 were collected in the Orbitrap, followed by data-dependent acquisition of MS/MS spectra for the 10 most abundant ions in the LTQ ion trap. Only ions having a charge state of ≥ +2 were considered for collision-induced dissociation in the ion trap. Repeated fragmentation of the same ion was minimized by employing a 30 second dynamic exclusion time.

MS Data Analysis Using Mascot: MS/MS spectra were searched against the Uniprot *Drosophila* protein database using the Mascot algorithm, version 2.3.2, provided by Matrix Science [41]. The Uniprot *Drosophila* database contained 37,560 protein entries (50% forward and 50% reversed, for FDR calculation). Mascot searches were performed with the following parameters: trypsin enzyme specificity, two possible missed cleavages, 20 ppm mass tolerance for precursor ions, and 0.5 Da mass tolerance for fragment ions. Search parameters specified a variable modification of oxidation on methionine and a static modification of carbamidomethylation (+57.0215 Da) on cysteine. To provide high confidence in peptide sequence assignment and protein identification, data were filtered following stringent criteria: Mowse score of > 28 for all charge states, at least two peptides per protein, 1% peptide false discovery rate (FDR), and 1% protein FDR.

#### Analysis of mass spectrometry and generation of enrichment score

For each protein identified from mass spectrometry, we averaged the number of uniquely identified peptides from two independent biological replicates. Since larger proteins have a greater number of unique peptides capable of being produced, we normalized the number of uniquely identified peptides by the length in amino acids of the protein. We also normalized the number of uniquely identified peptides by molecular weight and obtained comparable results. Next, we calculated enrichment of a protein over the negative IgG immunoprecipitation control by subtracting the length normalized unique peptide score from the IgG sample from the score obtained from the CLAMP immunoprecipitation sample. We then determined which proteins were present in both the S2 and Kc samples (S4 Table). Code for reproducing the mass spectrometry analysis is available on Github:

(https://github.com/JohnUrban/ClampMassSpec2016).

### Co-immunoprecipitation of IgG, CLAMP and NELF-B

Protein lysates for immunoprecipitation of IgG, CLAMP and NELF-B were prepared from *Drosophila* S2 and Kc cells following the non-crosslinked immunoprecipitation protocol outlined above. To immunoprecipitate NELF-B, we prepared anti-NELF-B Dynabeads using the Dynabeads Antibody coupling kit (ThermoFisher Scientific). Interactions between CLAMP and NELF-A/-B were detected using immunopreciptated eluates by western blotting (described below).

### Peak overlap analysis for CLAMP, GAF, and NELF

The data sets used for peak overlap analyses are all available through NCBI Gene Expression Omnibus (GEO). These data were all derived from the same cell type (*Drosophila* S2 modENCODE cells) and are as follows: CLAMP ChIP-seq: GSE39271, GAF ChIP-seq: GSE40646, NELF ChIP-chip: GSE20471. GAF peaks were used from GSE40646. For CLAMP ChIP-seq, peaks were called using the SPP software package [42] with the following parameters: window size = 150 and z = 7. Singular positions with very high tag counts were removed for the window size. To define NELF peaks, genomic regions with the fold-enrichment higher than 3 were used. Overlapping CLAMP, GAF and NELF peaks were identified and categorized as centered within 250bp of the transcription state site (TSS), between 250bp from the TSS and annotated transcription termination site (gene body), or otherwise in the intergenic region.

### Generation of dsRNA and RNAi treatment

Generation of dsRNA targeting *gfp* (control) and *clamp* RNAi has been previously validated and described in detail [5,6,11]. Three biological replicates of S2 cells were treated with 135μg of either *gfp* or *clamp* dsRNA following the previously described protocol [11].

### Chromatin Immunoprecipitation of NELF-B at promoters

The chromatin immunoprecipitation protocol following *gfp* and *clamp* RNAi has been previously described in detail [11]. In total, three biological replicates were prepared for ChIP following RNAi treatment. NELF-B ChIP was performed according to the published protocol with the modification that 2uL of the NELF-B antibody (rabbit, gift from Karen Adelman) was used per 1mL of chromatin.

### Quantitative real-time PCR for analysis of NELF-B enrichment

Quantification of NELF-B enrichment to promoters was performed using qPCR using a protocol that has been previously published and described in detail [11]. Primer sequences for promoter regions were used from previous publications [11,43]. We plotted the average enrichment values from three biological replicates with error bars representing +/- standard error of the mean. To test for significance, a Kruskal-Wallis ranks test was performed.

### Quantification of transcript abundance and western blotting

After treatment of S2 cells with either *clamp* or *gfp* RNAi, we collected a total of 2mL of cells for total RNA (1mL cells) and protein extraction (1mL cells). The preparation of mRNA for qPCR analysis was performed as previously described [7,11], with the exception that *gapdh* was used for internal normalization. Primers used to target amplification have been published previously [7,31]. The average ΔCt values for *clamp* or *nelf-b* transcript was calculated from four biological replicates and significant differences between means were calculated using a T-test.

Total protein was extracted to determine NELF-B abundance after *clamp* RNAi following the protocol described previously [7,11]. Immobilized proteins were blotted for NELF-B (rabbit, 1:1000, gift from Karen Adelman) and detected using the Western Breeze kit (ThermoFisher Scientific). A similar protocol was followed to detect associations between CLAMP and NELF-B after immunoprecipitation. For the detection of NELF-A, proteins were transferred to PVDF membrane using the Xcell II^TM^ blot module. The Western Breeze kit was then used to detect NELF-A (rabbit, 1:1000, gift from David Gilmour).

## Supporting information

S1 Fig Whole immuno-blot image of CLAMP and NELF-B immunoprecipitations. Either CLAMP or NELF-B was immunoprecipitated and samples were immuno-blotted for both NELF-A (A) and NELF-B (B) subunits of the NELF complex. CLAMP associates with both NELF subunits in male (S2, left column) and female (Kc, right column) cells, indicating a likely interaction with the entire NELF complex. The boxes on each blot indicate the cropped-area used in Fig 2.
(PDF)

S1 Table List of proteins identified only in Kc cells when comparing across the three mass spectrometry approaches. The asterisk indicates proteins with more than one isoform identified. While 102 proteins with multiple isoforms were identified, the number of proteins not including isoforms totals 50.
(PDF)

S2 Table Proteins identified either only in S2 cells (non-crosslinked) or cross-linked S2 cells. Twenty-nine proteins with multiple isoforms were identified in S2 cells not treated with cross-linking, however after removing multiple isoforms only 14 remained. For S2 cells that underwent cross-linking treatment, 55 total proteins were identified, with 28 remaining after removing multiple isoforms. Proteins with more than one isoform identified are indicated by the asterisk.
(PDF)

S3 Table Comparison of proteins identified in Kc and S2 cells, Kc and S2 cross-linked, and S2 with S2 cross-linked. Listed are the proteins found in common to two of the cells type data sets. The cross-linked S2 sample is abbreviated to S2XL. The asterisk marks proteins where multiple isoforms were identified.
(PDF)

S4 Table Enrichment scores for proteins identified in all three sample types. Listed are the names and enrichment scores for proteins identified in all three conditions. Enrichment was determined by dividing the number of uniquely identified peptides by the length in amino acids of the protein. Next, enrichment over the negative IgG control was calculated by subtracting the length normalized unique peptide score in the IgG sample from the score obtained from the CLAMP immunoprecipitation sample. Proteins listed with multiple isoforms identified are indicated by the asterisk.
(PDF)

S5 Table Percentages of peak overlap between CLAMP, GAF and NELF. Listed is the percentage of CLAMP (top), GAF (middle) or NELF (bottom) peaks that overlap with the indicated factors. Peaks marked as “not considered,” indicates that the presence or absence of the other protein was not taken under consideration. The first column (All) shows the percentages of peaks without taking into consideration genomic location. The last three columns indicate whether the peak is located within 250bp centered on the transcription start site (TSS), within the gene body (GB, measured from +250bp of the TSS to transcription termination site), or intergenic (all else).
(PDF)
