# Supplementary figures and images for: The Drosophila CLAMP protein associates with diverse proteins on chromatin
Source: PLoS One. 2017 Dec 27;12(12):e0189772. doi: 10.1371/journal.pone.0189772 (PMC5744976; doi:10.1371/journal.pone.0189772)

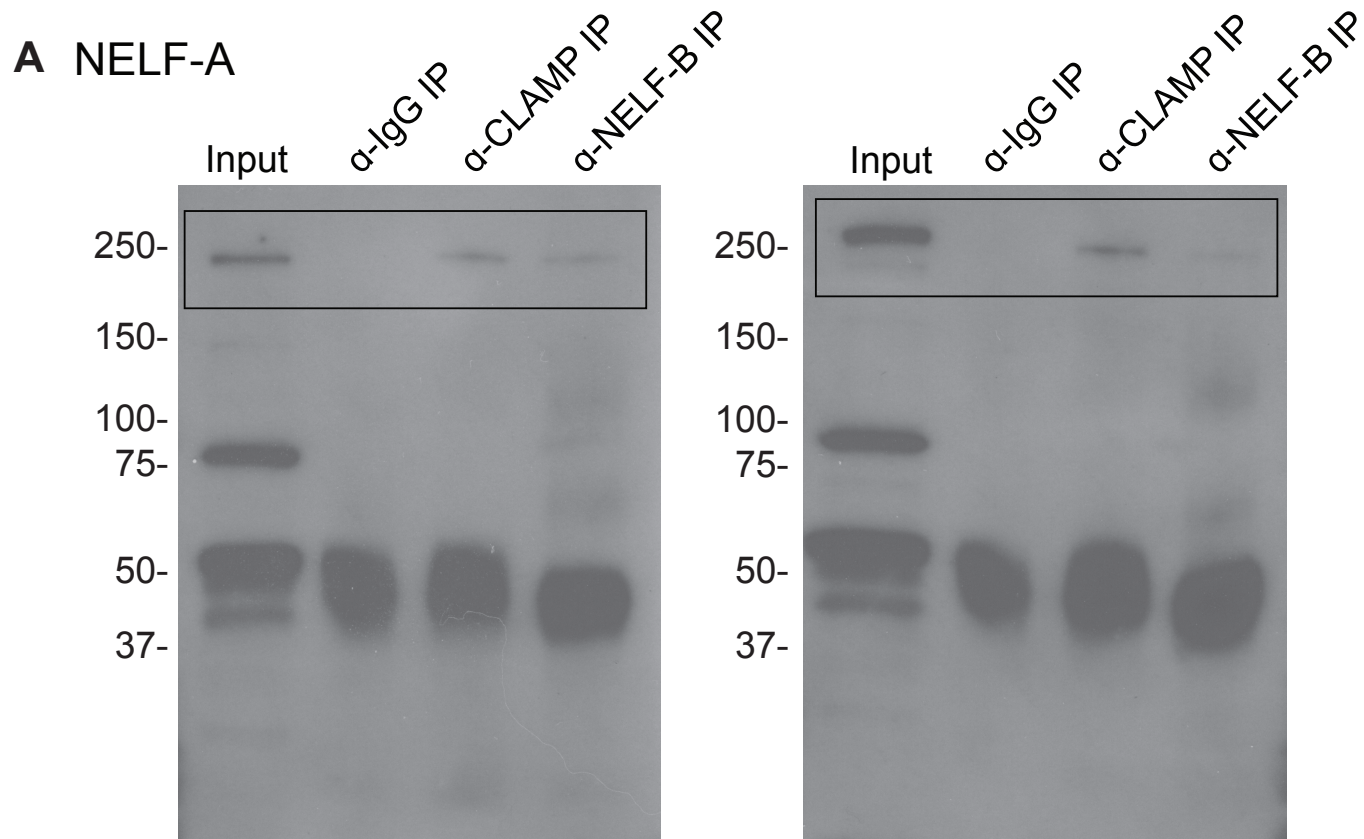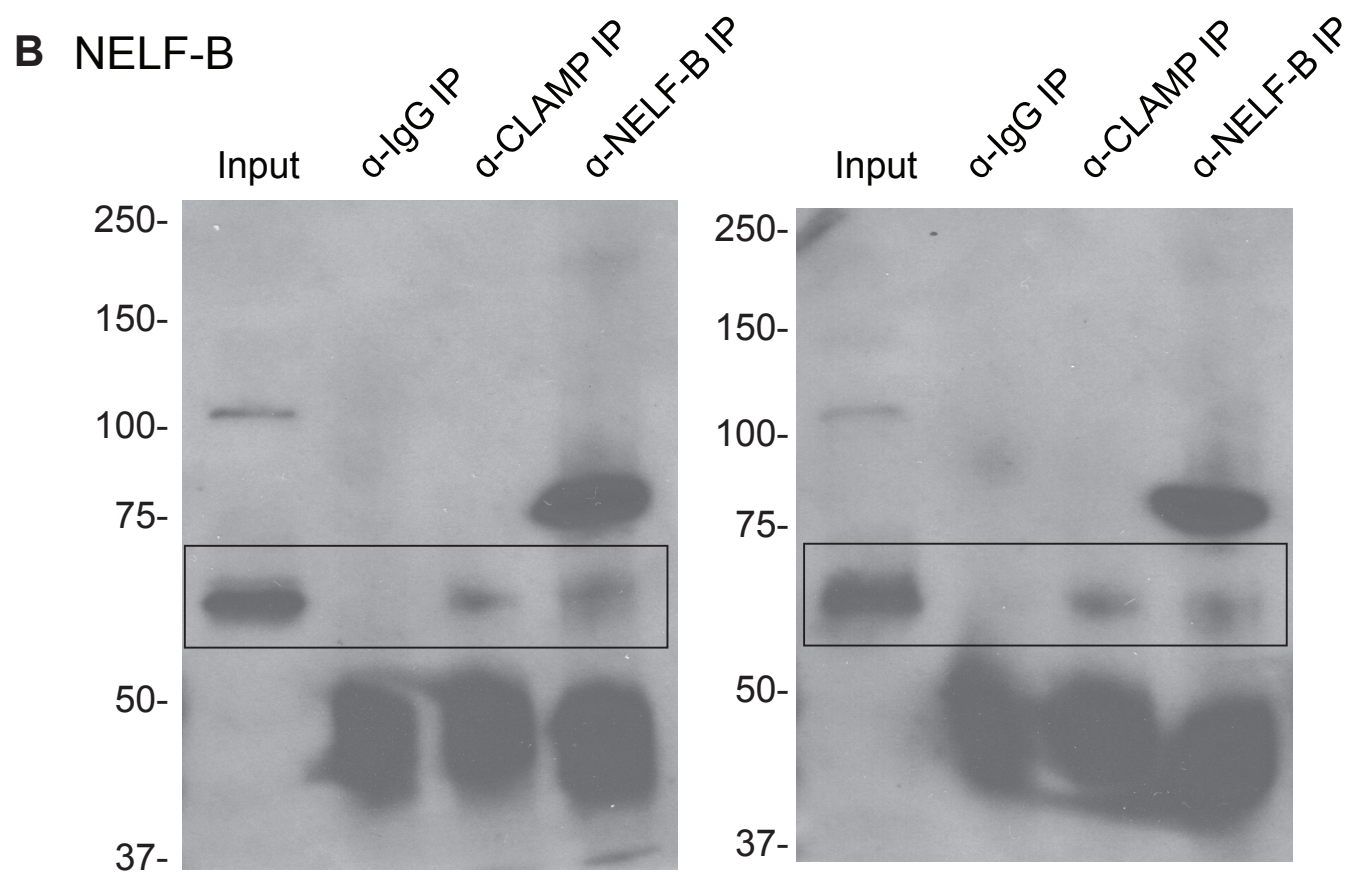

Male (S2)

Female (Kc)

Supplement: S1 Fig — Either CLAMP or NELF-B was immunoprecipitated and samples were immuno-blotted for both NELF-A (A) and NELF-B (B) subunits of the NELF complex. CLAMP associates with both NELF subunits in male (S2, left column) and female (Kc, right column) cells, indicating a likely interaction with the entire NELF complex. The boxes on each blot indicate the cropped-area used in Fig 2. (PDF) [file pone.0189772.s001.pdf]
