# Supplementary material for: The Drosophila CLAMP protein associates with diverse proteins on chromatin
Source: PLoS One. 2017 Dec 27;12(12):e0189772. doi: 10.1371/journal.pone.0189772 (PMC5744976; doi:10.1371/journal.pone.0189772)
Supplement: S1 Table — The asterisk indicates proteins with more than one isoform identified. While 102 proteins with multiple isoforms were identified, the number of proteins not including isoforms totals 50. (PDF) [file pone.0189772.s002.pdf]

S1 Table (Urban)

Kc Cells Protein Names	
ATP Synthase Beta subunit	Succinyl coenzyme A synthetase α subunit
Squid*	Dipeptidyl peptidase 3*
Bicaudal	Eukaryotic initiation factor 4A-III*
Mitochondrial trifunctional protein α subunit*	CG10166
Ran*	Sterol carrier protein X-related thiolase
Protein disulfide-isomerase	CG17597
delta-1-Pyrroline-5-carboxylate dehydrogenase 1	CG5776
CG10576	60 kDa heat shock protein homolog 2
Dihydrolipoyl dehydrogenase	Capulet*
Heterogeneous nuclear ribonucleoprotein at 98DE*	CG2076
60 kDa heat shock protein	Chd64*
Apontic*	Knockdown*
Heterogeneous nuclear ribonucleoprotein A1*	CG7033*
mitochondrial single stranded DNA-binding protein	Kayak
CG5044*	CG2918*
Thioredoxin peroxidase 2	Cyclophilin 1
calcium-binding protein 1	Ribonucleoside-diphosphate reductase large subunit
Short Stop*	ERp60*
CG5174*	Zipper*
Non-Specific Lethal 1*	CG8036*
Pyruvate kinase*	Sec61 α subunit
Heat shock 70 kDa protein cognate 5	Putative Achaete Scute Target 1*
Proteasome subunit beta type-1	Ecdysone-inducible gene L3
Failed axon connections*	Aldehyde dehydrogenase
p53*	NudC
