# Supplementary material for: The Drosophila CLAMP protein associates with diverse proteins on chromatin
Source: PLoS One. 2017 Dec 27;12(12):e0189772. doi: 10.1371/journal.pone.0189772 (PMC5744976; doi:10.1371/journal.pone.0189772)
Supplement: S2 Table — Twenty-nine proteins with multiple isoforms were identified in S2 cells not treated with cross-linking, however after removing multiple isoforms only 14 remained. For S2 cells that underwent cross-linking treatment, 55 total proteins were identified, with 28 remaining after removing multiple isoforms. Proteins with more than one isoform identified are indicated by the asterisk. (PDF) [file pone.0189772.s003.pdf]

S2 Table (Urban)

S2 Cells Protein Names	Crosslinked S2 Cells Protein Names	
Heterogeneous nuclear ribonucleoprotein at 27C*	Protein on ecdysone puffs*	Small ubiquitin-related modifier
Twenty-four*	Nup154*	Glutamyl-prolyl-tRNA synthetase*
Ataxin*	CG10077*	FK506-binding protein 1
Argonaute 2	Dynein heavy chain 64C*	Inner nuclear membrane protein Man1
CG17233*	Fmr1*	Elongation factor 2
Protein associated with topo II related-1	Ribosomal protein L30	Coatomer subunit gamma*
CG7239	Combgap*	Alan Shepard
CG42389*	Fibrillarin	CG9253
Serpent*	Ribosomal protein L26	putzig
Homeodomain interacting protein kinase	Lamin	nop5
Pumilio*	CG4747	14-3-3 protein zeta*
Caf1	Rm62*	Selenide,water dikinase
Myopic	Tudor staphylococcal nuclease	Growl*
Vacuolar protein sorting 60	Ribosomal protein S9	Nop56
