# Supplementary material for: The Drosophila CLAMP protein associates with diverse proteins on chromatin
Source: PLoS One. 2017 Dec 27;12(12):e0189772. doi: 10.1371/journal.pone.0189772 (PMC5744976; doi:10.1371/journal.pone.0189772)
Supplement: S3 Table — Listed are the proteins found in common to two of the cells type data sets. The cross-linked S2 sample is abbreviated to S2XL. The asterisk marks proteins where multiple isoforms were identified. (PDF) [file pone.0189772.s004.pdf]

Kc and S2 Cells	Kc and S2XL Cells	S2 and S2XL Cells
Nocte	Prohibitin*	Eukaryotic Translation Initiation Factor 4G*
Lingerer	lethal (2) 37Cc*	Glycoprotein 210 kDa
Bor	Ribosomal protein L6	Pif1A*
Uridine_kinase*	Histone H3*	Histone 4
Calreticulin	Histone H3.3	Ribosomal protein L23A
Histone H2A*	Quaking Related *	
Porin	Modulo	
