# Supplementary material for: The Drosophila CLAMP protein associates with diverse proteins on chromatin
Source: PLoS One. 2017 Dec 27;12(12):e0189772. doi: 10.1371/journal.pone.0189772 (PMC5744976; doi:10.1371/journal.pone.0189772)
Supplement: S4 Table — Listed are the names and enrichment scores for proteins identified in all three conditions. Enrichment was determined by dividing the number of uniquely identified peptides by the length in amino acids of the protein. Next, enrichment over the negative IgG control was calculated by subtracting the length normalized unique peptide score in the IgG sample from the score obtained from the CLAMP immunoprecipitation sample. Proteins listed with multiple isoforms identified are indicated by the asterisk. (PDF) [file pone.0189772.s005.pdf]

Protein Name	S2 Cells	Kc Cells	S2 Cells XL
**CLAMP***	10.250	8.913	160.107
**Stress-Sensitive B***	6.689	16.722	-209.431
**NOCTE**	4.223	3.573	-8.896
**Elongation factor 1-alpha***	2.706	1.623	122.121
**Histone H2B**	2.033	4.065	-1284.309
**NELF-A**	1.199	2.398	41.551
**Syncrip***	0.945	0.473	38.658
**Porin**	0.887	5.319	-175.284
**Bor**	0.828	1.656	-259.205
**Calreticulin**	0.616	1.847	0.000
**Lingerer***	0.563	0.188	0.000
**Uridine kinase***	0.446	0.446	0.000
**Clueless***	0.173	0.863	47.935
