# Supplementary material for: The Drosophila CLAMP protein associates with diverse proteins on chromatin
Source: PLoS One. 2017 Dec 27;12(12):e0189772. doi: 10.1371/journal.pone.0189772 (PMC5744976; doi:10.1371/journal.pone.0189772)
Supplement: S5 Table — Listed is the percentage of CLAMP (top), GAF (middle) or NELF (bottom) peaks that overlap with the indicated factors. Peaks marked as “not considered,” indicates that the presence or absence of the other protein was not taken under consideration. The first column (All) shows the percentages of peaks without taking into consideration genomic location. The last three columns indicate whether the peak is located within 250bp centered on the transcription start site (TSS), within the gene body (GB, measured from +250bp of the TSS to transcription termination site), or intergenic (all else). (PDF) [file pone.0189772.s006.pdf]

S5 Table (Urban)

	All	TSS+/-250	GB (TSS+250:TTS)	Intergenic (rest)
All CLAMP peaks		31.6	51.1	17.3
CLAMP only	54.38632619	37.3	42.2	20.5
CLAMP with only GAF	34.81603515	16.7	67.5	15.8
CLAMP with only NELF	2.169137836	72.8	18.4	8.9
CLAMP with GAF and NELF	8.628500824	45.9	48.9	5.2
CLAMP with GAF (NELF is not considered)	43.44453597	22.51540528	63.81734871	13.66724601
CLAMP with NELF (GAF is not considered)	10.79763866	51.30324221	42.78448824	5.912269549
All GAF peaks		18.4	65.7	15.9
GAF only	17.91685495	16.4	61.4	22.2
GAF with only CLAMP	61.65838229	14.8	69.5	15.8
GAF with only NELF	0.338906462	33.3	46.7	20
GAF with CLAMP and NELF	20.0858563	30.9	58.3	10.8
GAF with CLAMP (NELF is not considered)	81.74423859	18.73963516	66.72194583	14.53841902
GAF with NELF (CLAMP is not considered)	20.42476277	30.97345133	58.07522124	10.95132743
ALL NELF peaks		43.4	47.8	8.8
NELF only	18.1773399	16	59.6	24.4
NELF with only CLAMP	16.10837438	70.9	20.5	8.6
NELF with only GAF	9.458128079	18.2	77.6	4.2
NELF with CLAMP and GAF	56.25615764	48.6	46.8	4.6
NELF with CLAMP (GAF is not considered)	72.36453202	53.57385977	40.91218516	5.513955071
NELF with GAF (CLAMP is not considered)	65.71428571	44.22788606	51.1994003	4.572713643
